# Supplementary material for: Intracellular localisation of Mycobacterium tuberculosis affects efficacy of the antibiotic pyrazinamide
Source: Nat Commun. 2021 Jun 21;12:3816. doi: 10.1038/s41467-021-24127-3 (PMC8217510; doi:10.1038/s41467-021-24127-3)
Supplement: Supplementary file 1 — Supplementary Information [file 41467_2021_24127_MOESM1_ESM.pdf]

Supplementary Information

Intracellular localisation of *Mycobacterium tuberculosis* affects efficacy of the antibiotic pyrazinamide

Pierre Santucci[1], Daniel J. Greenwood[1§], Antony Fearns[1], Kai Chen[2], Haibo Jiang[2,3]* and Maximiliano G. Gutierrez[1]*

Affiliations:

[1]Host-Pathogen Interactions in Tuberculosis Laboratory, The Francis Crick Institute, 1 Midland Road, London, NW1 1AT, United Kingdom.

[2]School of Molecular Sciences, University of Western Australia, Perth, AU.

[3]Department of Chemistry, The University of Hong Kong, Hong Kong, China.

[§]Present address: Institute of Molecular Systems Biology, ETH, Zurich, Switzerland

*Correspondence to: max.g@crick.ac.uk and haibo.jiang@uwa.edu.au

This file contains the Supplementary Figures (S1-S14) and their respective legends

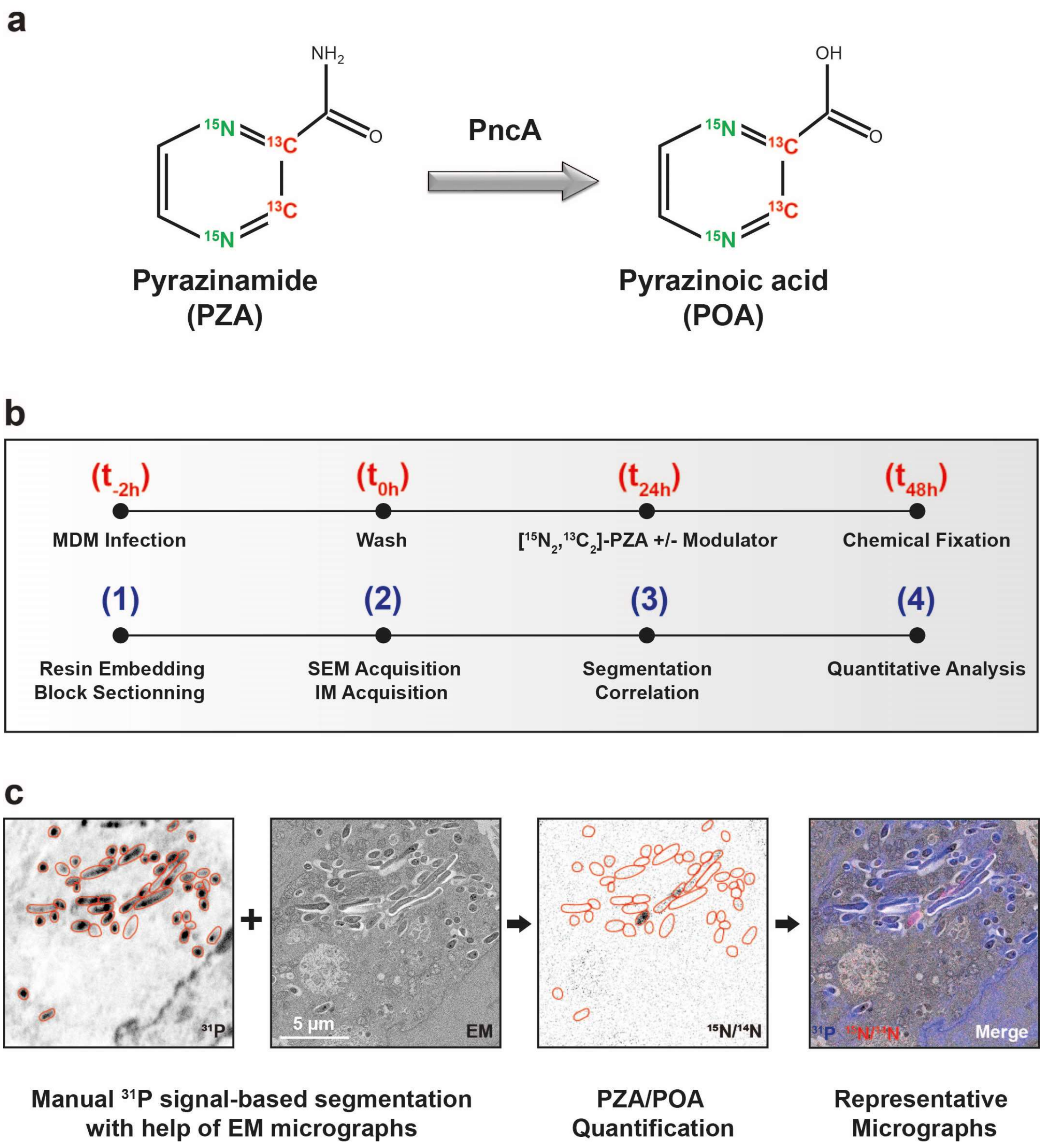

Supplementary Fig. 1 Experimental and analytical electron and ion microscopy workflow
(a) Chemical structures of PZA and POA containing isotopically labelled ^{13}C and ^{15}N atoms, displayed in red and green respectively. Conversion of the prodrug PZA into POA is mediated by the bacterial pyrazinamidase PncA. **(b)** Schematic representation of the experimental procedure followed to perform MDM infection, [$^{15}N_2$, $^{13}C_2$]-PZA treatment, electron/ion microscopy samples processing and images acquisition. **(c)** Schematic representation of the segmentation and analysis pipeline used in this study to perform quantitative analysis of ion microscopy micrographs. The complete procedures are detailed in the *Methods* section of this manuscript.

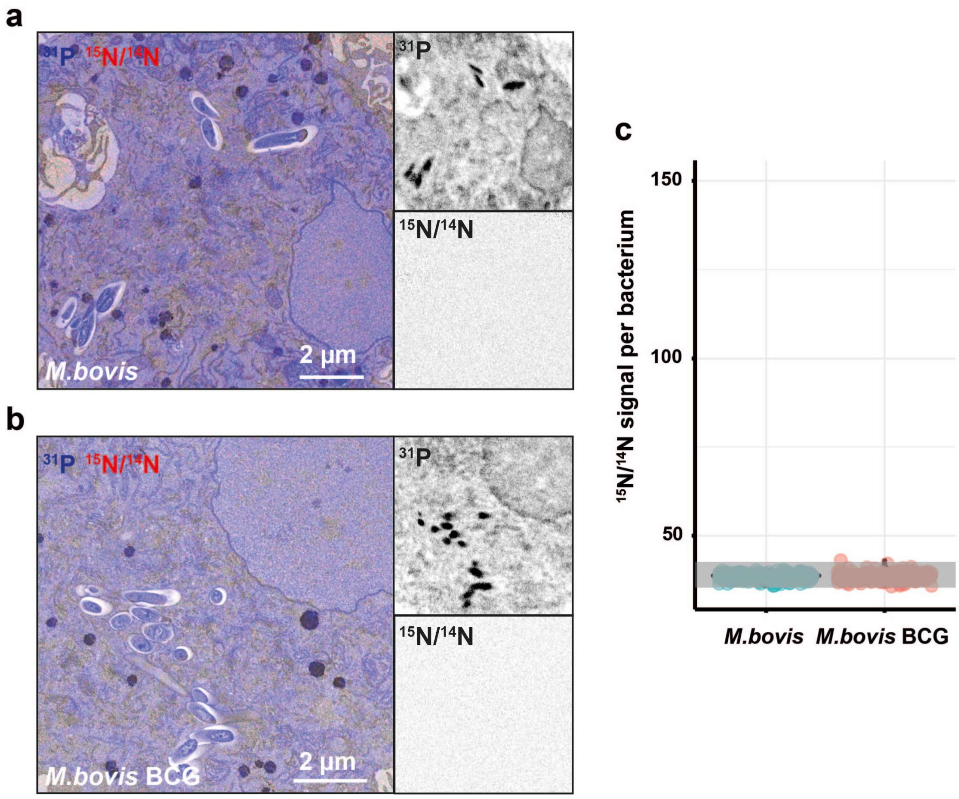

Supplementary Fig. 2 Functional pyrazinamidase activity is required for enrichment of $^{15}N/^{14}N$
(a-b) Representative images of PZA/POA distribution in intracellular *M. bovis* and *M. bovis* BCG strains. MDM were infected with *M. bovis* or *M. bovis* BCG and treated with 30 mg/L [$^{15}N_2$, $^{13}C_2$]-PZA for 24 hours. EM micrographs are overlaid with ^{31}P (blue) and $^{15}N/^{14}N$ (red) NanoSIMS images. Magnifications show ^{31}P (top panel) and $^{15}N/^{14}N$ (bottom panel) individual images at the single bacterial-cell level. Scale bars correspond to 2 µm. Micrographs are representative of 2 independent experiments. **(c)** Quantitative analysis of $^{15}N/^{14}N$ signal per bacterium shown as violin plot with single dots. Grey line indicates the natural background level of the $^{15}N/^{14}N$ enrichment. Results were obtained from 227-298 individually segmented bacteria from n = 2 biologically independent experiments.

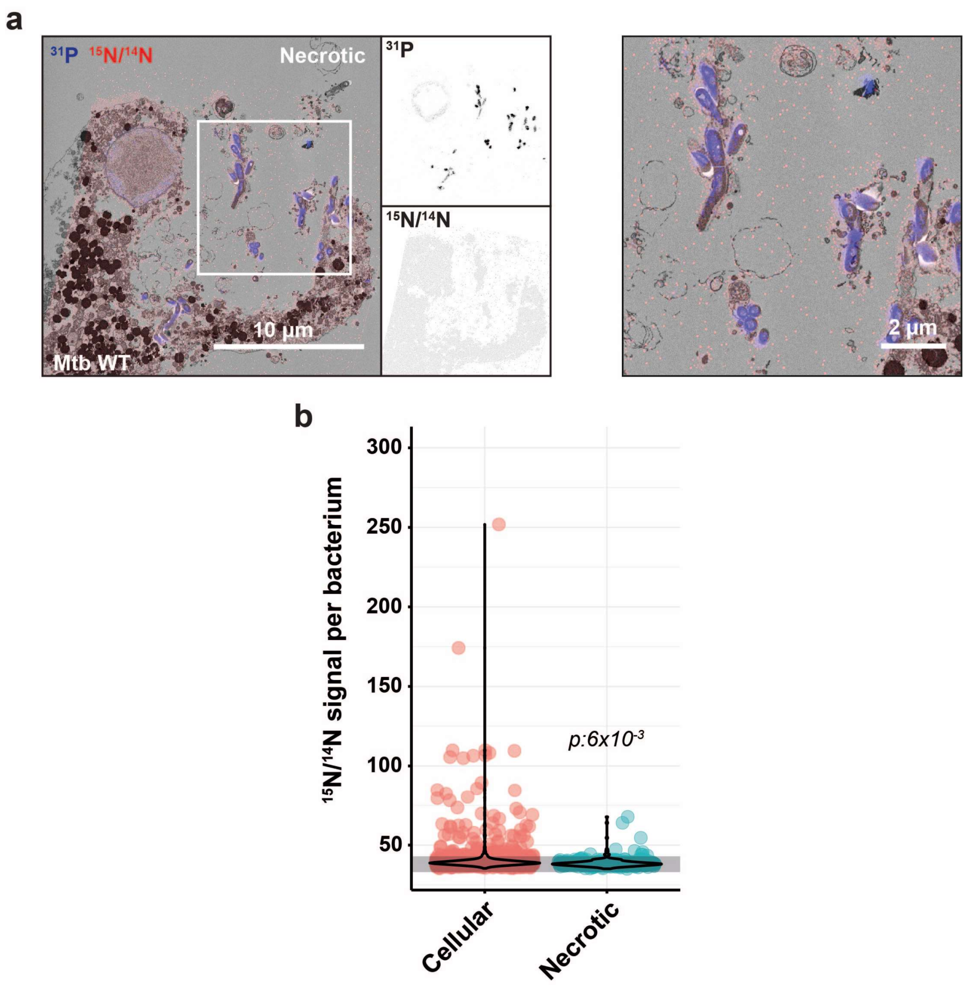

Supplementary Fig. 3 Plasma membrane integrity of infected cells contribute to PZA/POA accumulation in Mtb
(a) Representative images of PZA/POA distribution Mtb WT contained within a necrotic cell. Mtb-infected MDM were treated with 30 mg/L [$^{15}N_2$, $^{13}C_2$]-PZA for 24 hours. EM micrograph is overlaid with ^{31}P (blue) and $^{15}N/^{14}N$ (red) NanoSIMS images. Magnifications show ^{31}P (top panel) and $^{15}N/^{14}N$ (bottom panel) individual images at the single bacterial-cell level. Scale bar corresponds to 10 µm. Region of interest highlighted by the white rectangle, is shown in detail in the right panel. Scale bar corresponds to 2 µm. Micrographs are representative of 2 independent experiments. **(b)** Quantitative analysis of $^{15}N/^{14}N$ signal per bacterium within intact cells (Cellular) and necrotic (Necrotic) cells, shown as violin plot with single dots. Grey line indicates the background level of the ^{15}N enrichment. Results were obtained from 673 individually segmented bacteria from n = 2 biologically independent experiments and *p*-values were calculated by using a two-tailed t-statistic test from the linear model.

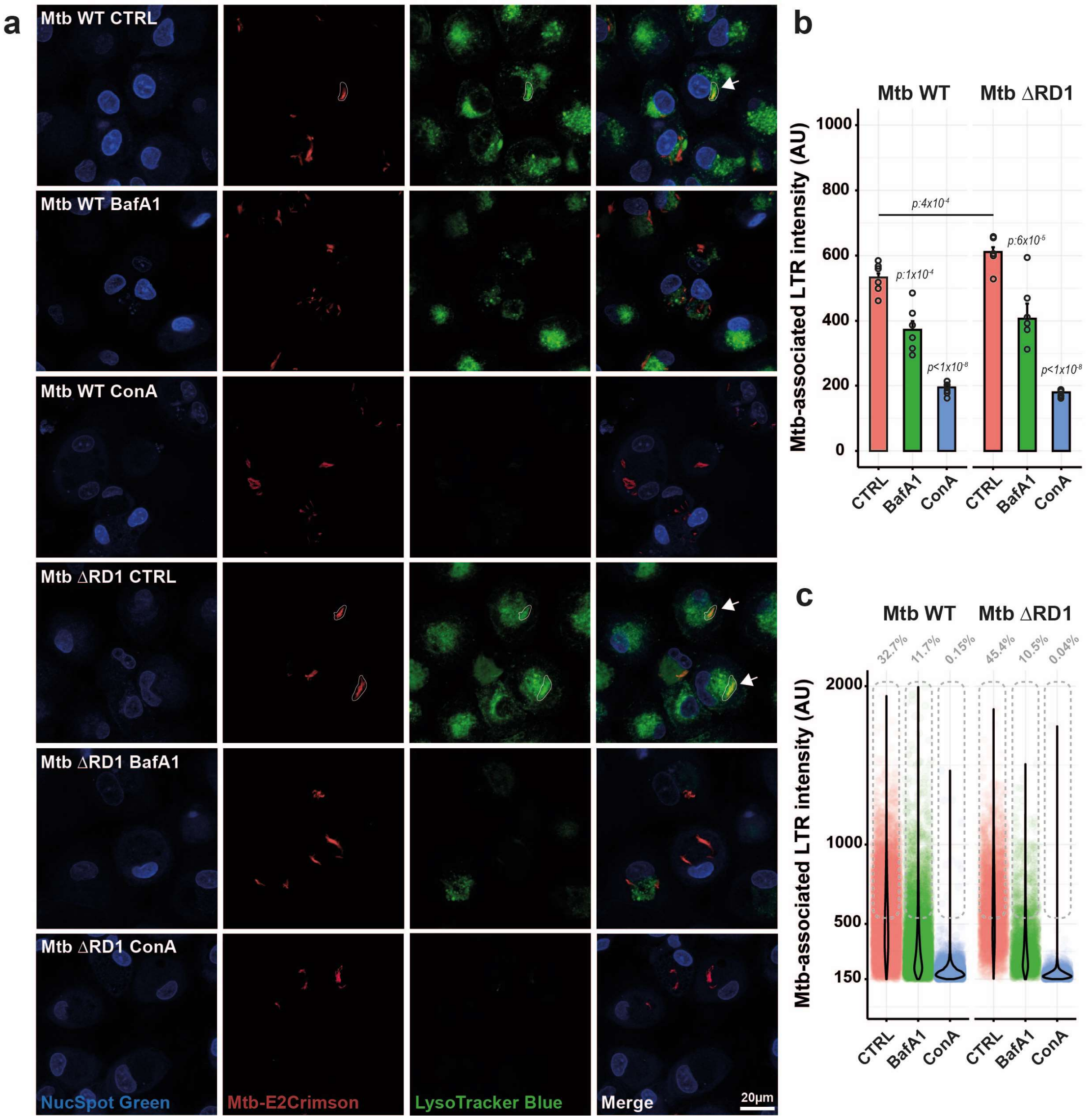

Supplementary Fig. 4 Intracellular Mtb ΔRD1 localises more often in acidic environments than Mtb WT
(a) Representative fluorescence images of Mtb WT and Mtb ΔRD1-infected MDM stained with LysoTracker in the presence or absence of BafA1 or ConA. Infected cells were exposed for 24 hours to 100 nM of BafA1, 100 nM ConA or left untreated, then cells were pulsed with 200 nM of LysoTracker Blue for 30 min and counter stained with NucSpot Green dye. Live cells were imaged live using the OPERA Phenix. Magnifications display nuclear staining (blue), Mtb E2-Crimson (red) and LysoTracker labelling (green). Scale bar corresponds to 20 µm. Region of interests showing Mtb-LysoTracker co-localization are highlighted by the white arrows. Micrographs are representative of 2 independent experiments. **(b)** Quantification of Mtb-associated LysoTracker mean fluorescence intensities. Results are expressed as mean ± SEM from n = 2 biologically independent experiments performed at least in three technical replicates. Statistical significance in comparison to the respective control condition or between Mtb WT and Mtb ΔRD1 was assessed by comparing the means of each conditions using one-way ANOVA followed with Tukey's multiple comparisons test. **(c)** Quantitative analysis of LysoTracker mean fluorescence signal per bacterial region of interest shown as violin plot with single dots. The percentage of LysoTracker positive events is displayed on top of the grey boxes. Results for WT and ΔRD1 were obtained from 7200-10946 and 2542-3656 individually segmented bacterial regions of interest respectively, from n = 2 biologically independent experiments.

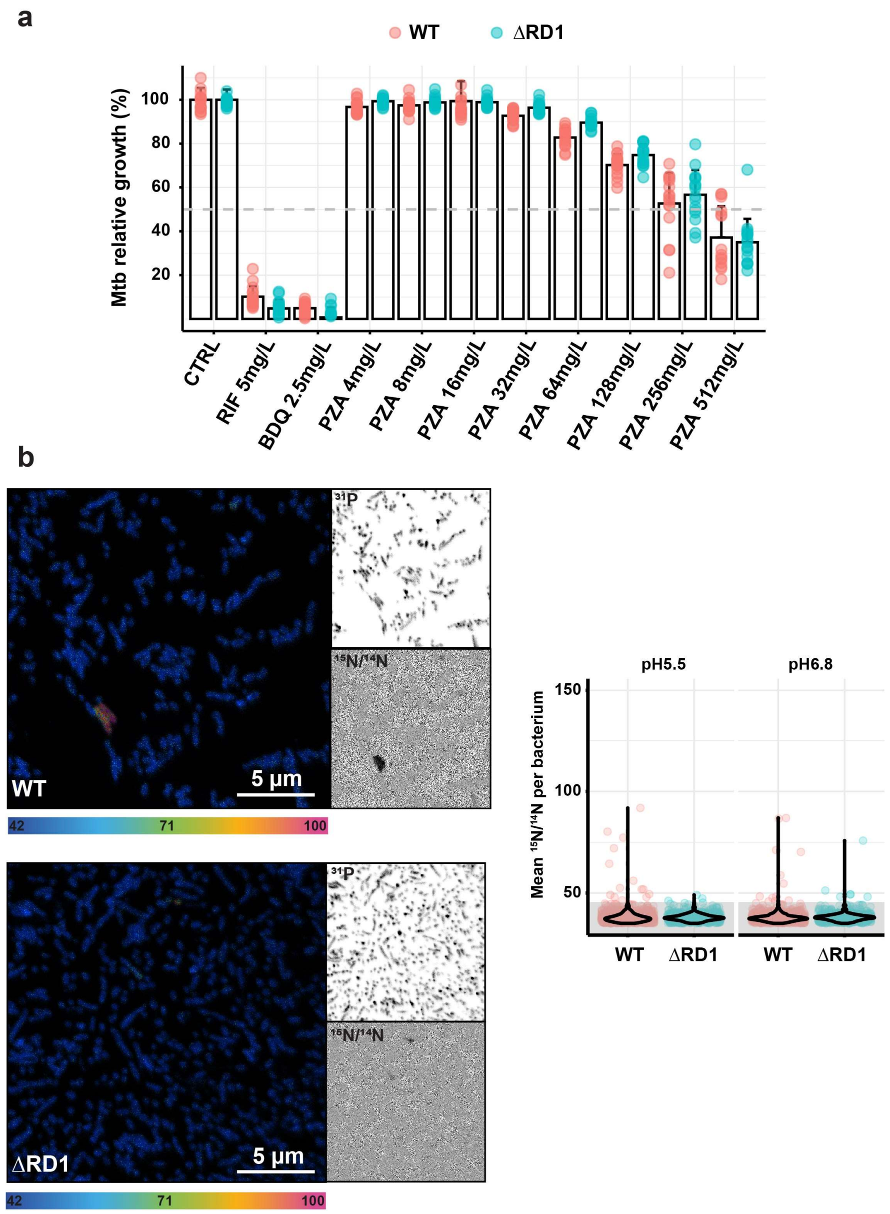

Supplementary Fig. 5 Mtb WT and Mtb ΔRD1 accumulates similar PZA/POA and display identical antibiotic susceptibility towards PZA *in vitro*

(a) The activity of PZA onto Mtb WT and Mtb ΔRD1 *in vitro* was tested using the microdilution methods. Susceptibility was assessed using increasing concentration of PZA ranging from 4 to 512 mg/L in complete Middlebrook 7H9 broth, and 5 µg/mL BDQ or 2.5 µg/mL RIF were used as positive inhibition control. After 14 days of incubation, plates were scanned and OD_{600nm} was determined. Results were expressed as mean relative growth ± SD where Mtb WT and Mtb ΔRD1 growth in complete Middlebrook 7H9 broth was considered as 100 %. Grey dashed line indicates 50% of inhibition. Experiments were from n = 2 biologically independent experiments with at least 4 to 6 technical replicates. **(b)** Representative images of PZA/POA accumulation in Mtb WT and Mtb ΔRD1 *in vitro* at pH6.8. Mtb strains were grown until reaching mid-exponentially growing phase in Middlebrook 7H9 medium, inoculated in fresh media adjusted at pH6.8 or pH5.5 and further treated with 30 mg/L [$^{15}N_2$, $^{13}C_2$]-PZA for 24 hours. Micrographs display $^{15}N/^{14}N$ signals as hyperspectral images. Scale bars correspond to 5 µm. Magnifications show ^{31}P (top panel) and $^{15}N/^{14}N$ (bottom panel) individual NanoSIMS images. On the right, the quantitative analysis of $^{15}N/^{14}N$ signal per bacterium shown as violin plot with single dots. Grey line indicates the natural background level of the $^{15}N/^{14}N$ enrichment. In that specific context, bacterial segmentation was performed using the ^{31}P signal and the “threshold” function from FIJI. The “analyse particles” function from FIJI was used to quantify $^{15}N/^{14}N$ in each region of interest. Results are from 919-1059 individual regions of interests from n = 1 biologically independent experiment.

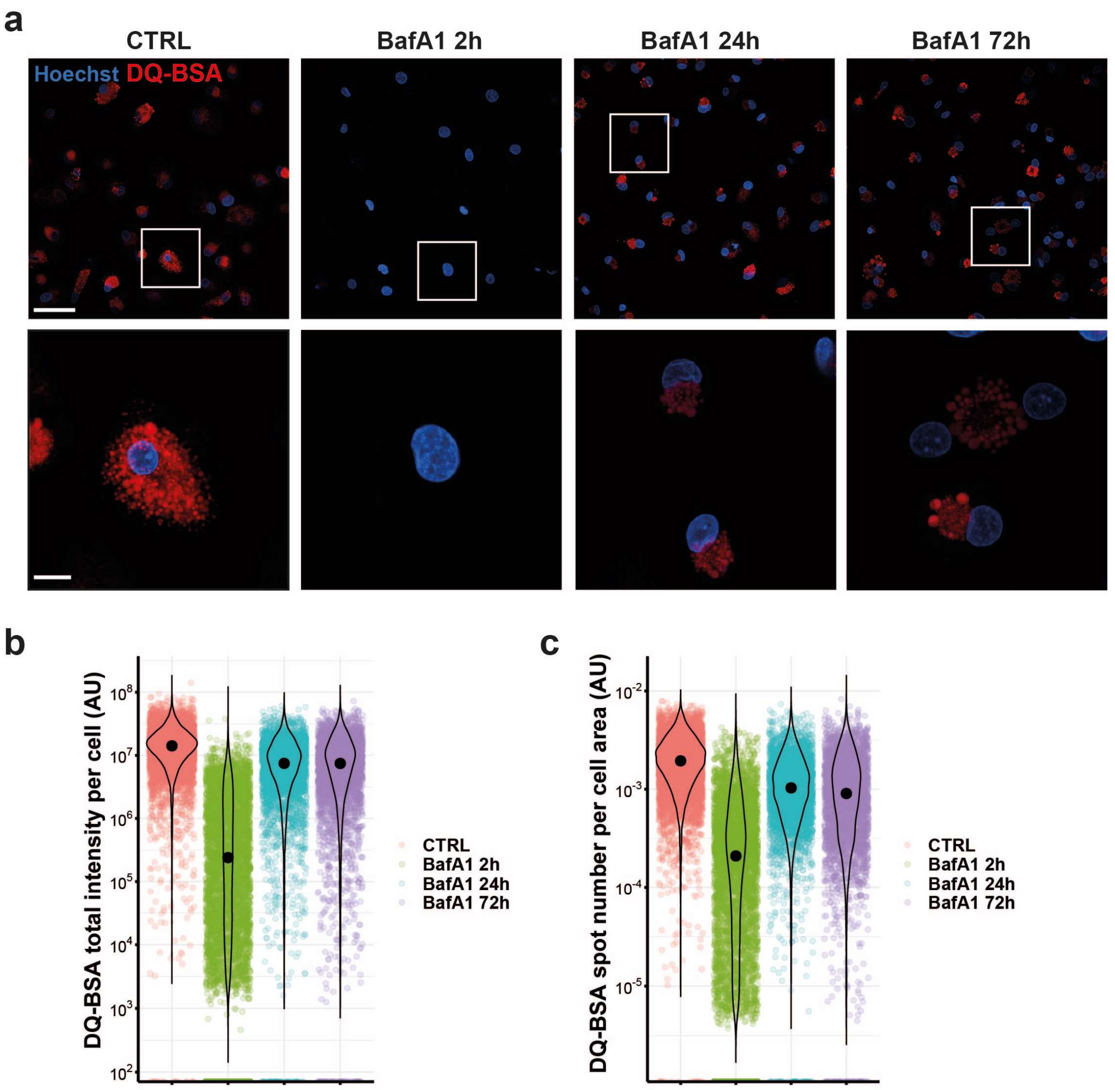

Supplementary Fig. 6 BafA1 treatment impairs endolysosomal proteolytic activity in MDM

(a) Representative fluorescence images of DQ-BSA stained MDM in the presence or absence of BafA1. Approximately 30,000 MDM were exposed for 2, 24 or 72 hours to 100 nM of BafA1 or left untreated, then cells were pulsed with 10 µg/ml of DQ™ Red BSA for 4 hours in the continuous presence of the modulator BafA1 (except the for the control). After 4 hours, cells were washed, stained with Hoechst and imaged live using the OPERA Phenix. Magnifications display nuclear staining (blue) and DQ-BSA labelling (red). Scale bar corresponds to 50 µm. Region of interests highlighted by the white rectangles, are shown in detail in the bottom panels respectively. Scale bar corresponds to 5 µm. Micrographs are representative of 2 independent experiments. **(b)** Quantitative analysis of DQ-BSA mean fluorescence intensity per cell expressed as arbitrary units (AU). **(c)** Quantitative analysis of DQ-BSA spot number normalized per cell area expressed as arbitrary units (AU). Analysis was performed using the “Find spot”, “Morphology properties” and “Intensity properties” building blocks from the Harmony software (Perkin Elmer, version 4.9). From 3420 to 7101 stained MDM were analysed. Results are from n = 2 biologically independent experiments.

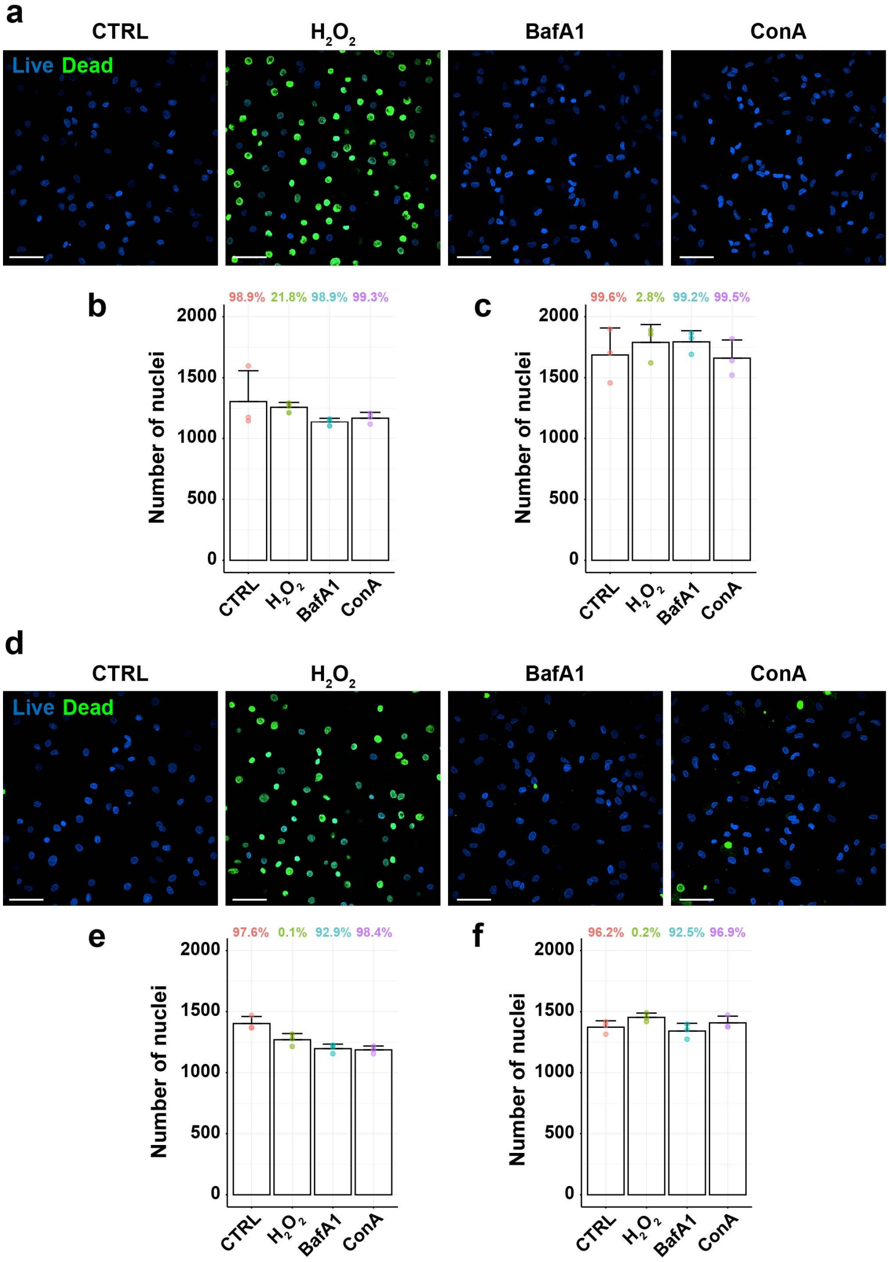

Supplementary Fig. 7 Inhibition of MDM endolysosomal acidification by BafA1 and ConA doesn't trigger any cytotoxic effects

(a) Representative fluorescence images of Blue/Green (Live/Dead) stained MDM in the presence or absence of v-ATPase inhibitors for 24 hours. Approximately 30,000 MDM were exposed for 24 hours to 100 nM BafA1, 100 nM ConA or left untreated. Hydrogen peroxide (50 mM) was used as positive control in this assay. Live-cell imaging was performed using the OPERA Phenix microscope with a 40x water-immersion objective. Magnifications display live nuclear staining (blue) and dead nuclear straining (green). Scale bar corresponds to 50 µm. Micrographs are representative of 2 independent experiments. **(b-c)** Quantification of the total number of nuclei detected (y-axis) in each condition after 24 hours. The percentage of green (dead) nuclei in each condition is display on top of each bar chart. Results are expressed as mean ± SD. Each panel represents n = 1 independent biological experiment performed in technical triplicate. **(d)** Representative fluorescence images of Blue/Green (Live/Dead) stained MDM in the presence or absence of v-ATPase inhibitors for 72 hours. Approximately 30,000 MDM were exposed for 72 hours to 100 nM BafA1, 100 nM ConA or left untreated. Micrographs are representative of 2 independent experiments and magnifications are displayed as described in **(a). (e-f)** Quantification of the total number of nuclei detected (y-axis) in each condition after 72 hours. The percentage of green (dead) nuclei in each condition is display on top of each bar chart. Results are expressed as mean ± SD. Each panel represents n = 1 independent biological experiment performed in technical triplicate.

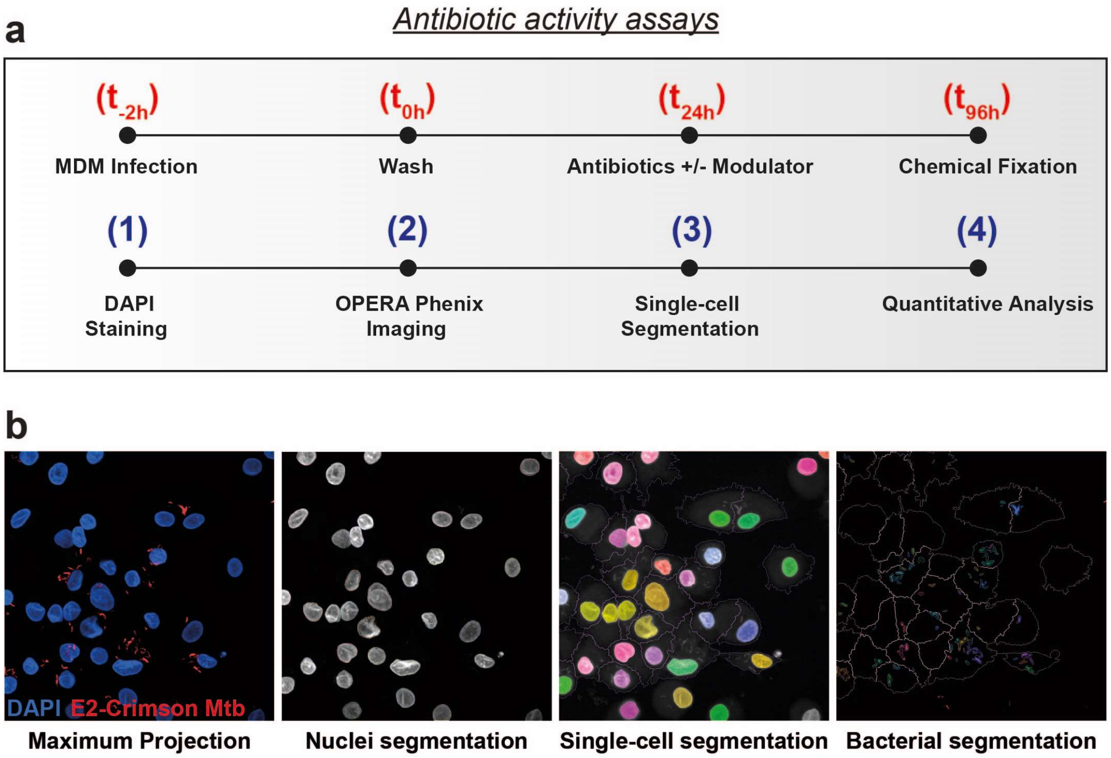

Supplementary Fig. 8 Experimental and analytical workflow of high-content intracellular antibiotic susceptibility assays.
(a) Schematic representation of the experimental procedure followed to perform MDM infection, antibiotic treatment, chemical fixation, staining, image acquisition and analysis. **(b)** Schematic representation of the segmentation and analysis pipeline used in this study to perform quantitative analysis of fluorescence microscopy micrographs. The complete procedures are detailed in the *Methods* section of this manuscript.

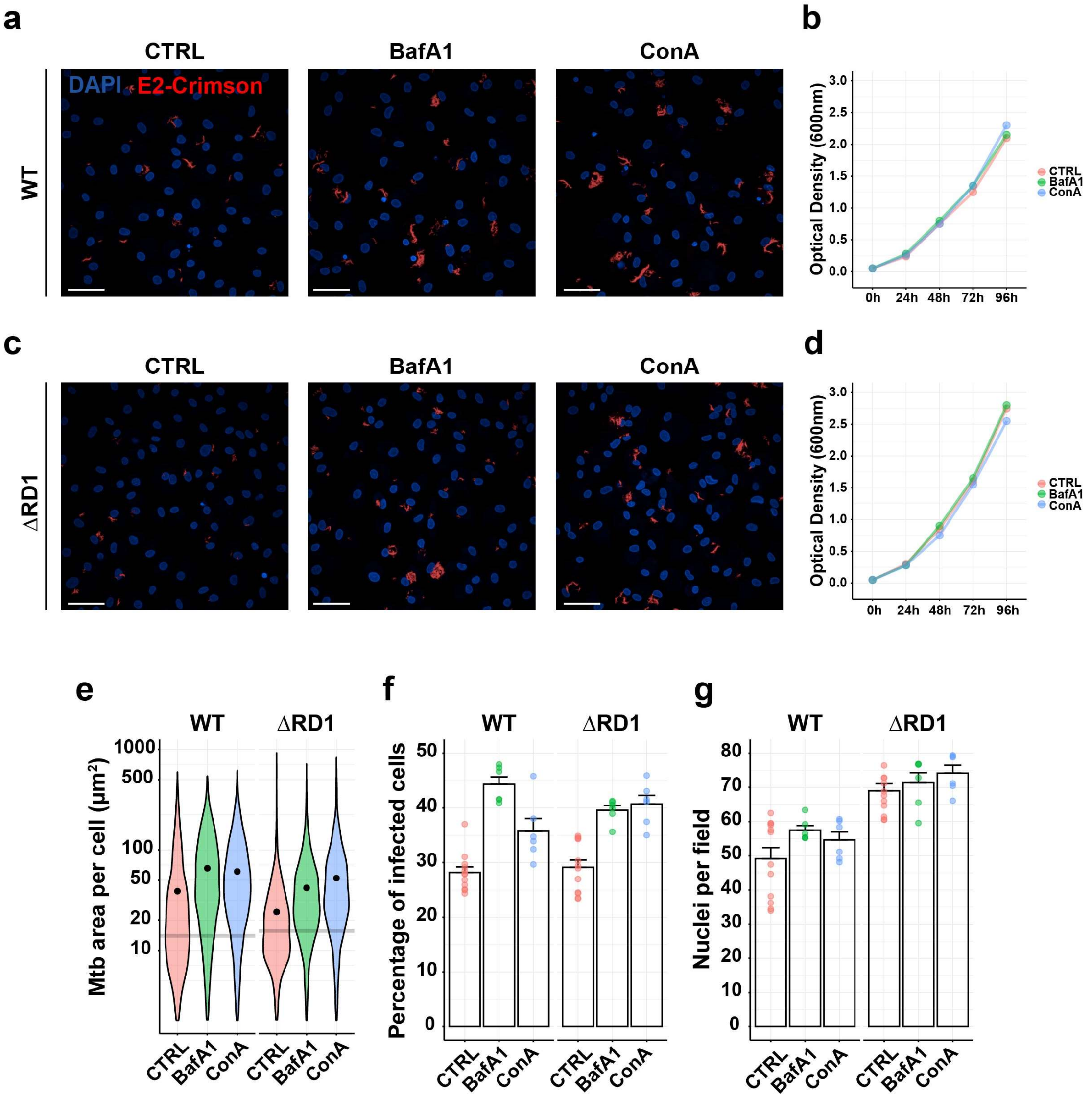

Supplementary Fig. 9 Inhibition of MDM endolysosomal acidification by BafA1 and ConA promotes Mtb replication and cell to cell spread.

(a and c) Representative confocal fluorescence images of Mtb WT and Mtb ΔRD1-infected MDM for 24 hours and further treated for 72 hours in the presence or absence of v-ATPase inhibitors. Magnifications display nuclear staining (blue) and Mtb-producing E2-Crimson (red). Scale bar corresponds to 50 µm. Micrographs are representative of 2 independent experiments. **(b and d)** Growth curves of Mtb WT and ΔRD1 strains in Middlebrook 7H9 medium in the presence of 100 nM BafA1, 100 nM ConA or left untreated. Representative experiment out of two biological replicates. **(e)** Quantitative analysis of E2-Crimson Mtb WT and Mtb ΔRD1 area per single-cell expressed in µm^2. Results are displayed in violin plots where grey lines represent the mean Mtb area per cell pre-treatment ($t_{24\,h}$) and black dots represent the mean Mtb area per cell post-treatment ($t_{96\,h}$). From 2124 to 2722 and 3273 to 4775 infected MDM were analysed for Mtb WT and Mtb ΔRD1 respectively. Results are from n = 2 biologically independent experiments. **(f)** Quantification of the mean proportion of Mtb WT and Mtb ΔRD1 infected cells in the presence or absence of v-ATPase inhibitors 96 h after infection. Results are expressed as mean ± SD. Results are from n = 2 biologically independent experiments with at least 3 to 6 technical replicates per condition. **(g)** Quantification of the average number of nuclei per field during Mtb WT and Mtb ΔRD1 infection in the presence or absence of v-ATPase inhibitors 96 h after infection. A fixed number of 35 fields were images using the OPERA Phenix microscope with a 40x water-immersion objective. Results are expressed as mean ± SEM. Results are from n = 2 biologically independent experiments with at least 3 to 6 technical replicates per condition.

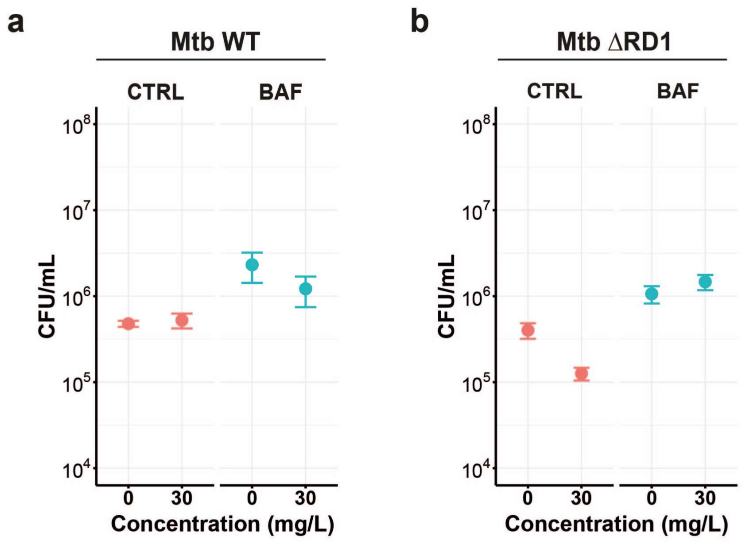

Supplementary Fig. 10 PZA inhibition is more effective against Mtb ΔRD1 mutant strain
(a-b) Intracellular replication of Mtb WT and Mtb ΔRD1 in the presence of PZA and 100 nM BafA1 assessed by CFU counting. MDM were infected for 24 hours and further treated for an additional 72 hours with/without 30 mg/L PZA in the presence or absence of v-ATPase inhibitor. Cells were lysed using PBS-Triton X100 0.1%, serially diluted and plated onto 7H11 agar plates. Plates were incubated at 37 °C for 4 weeks. Results are expressed as means of technical triplicate ± SEM. Representative experiment out of two biological replicates.

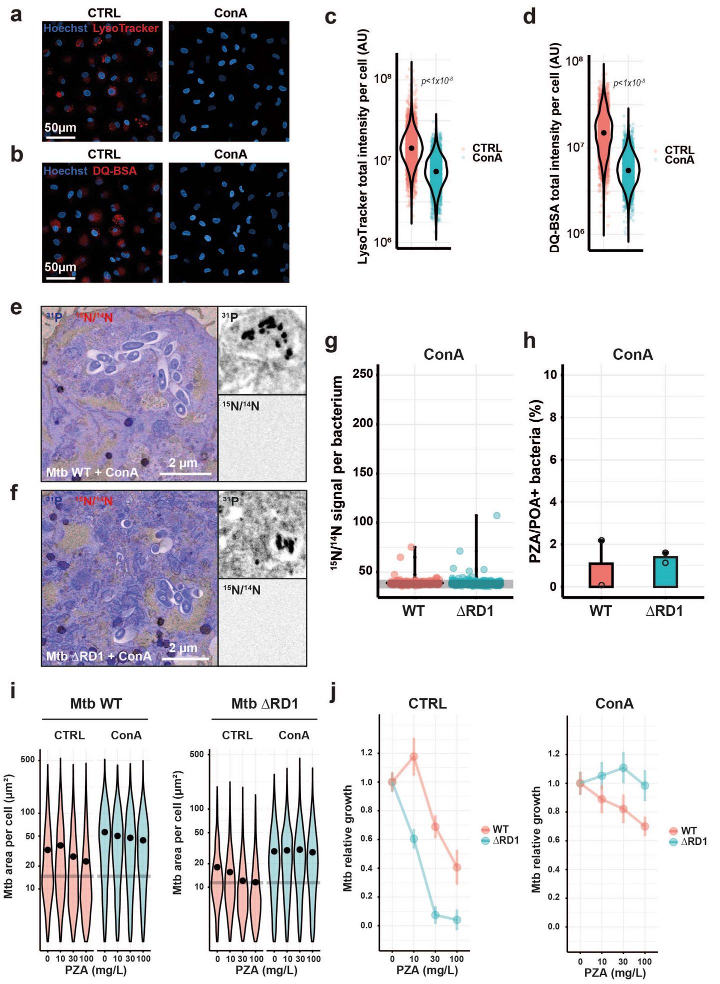

Supplementary Fig. 11 Inhibition of MDM endolysosomal acidification by ConA impairs PZA/POA accumulation and efficacy

(a-b) Representative fluorescence images of LysoTracker and DQ-BSA stained MDM in the presence or absence of ConA. Approximately 30,000 MDM were treated or not for 24 h with 100 nM ConA, then cells were stained with 200 nM LysoTracker Red for 30 min or pulsed with 10 µg/ml of DQ™ Red BSA for 4 h. After staining, cells were washed, stained with Hoechst and imaged live. Magnifications show nuclear staining (blue) and **(a)** LysoTracker or **(b)** DQ-BSA labelling (red). Scale bar corresponds to 50 µm. Micrographs are representative of 2 independent experiments. **(c-d)** Quantitative analysis of LysoTracker or DQ-BSA total fluorescence intensity per cell expressed as arbitrary units (AU). Results are expressed as violin plots where the mean is displayed as central black dot. Statistical significance was assessed with one-way ANOVA followed by a pairwise t-test using R. **(e-f)** Representative images of PZA/POA distribution in intracellular Mtb WT and Mtb ΔRD1 strains treated with ConA. MDM were infected with Mtb WT or Mtb ΔRD1 and treated with 30 mg/L [$^{15}N_2$, $^{13}C_2$]-PZA in the presence of 100nM of ConA for 24 h. EM micrographs are overlaid with ^{31}P (blue) and ^{15}N/^{14}N (red) NanoSIMS images. Magnifications show ^{31}P (top panel) and ^{15}N/^{14}N (bottom panel) individual images at the single bacterial-cell level. Scale bars correspond to 2 µm. Micrographs are representative of 2 independent experiments. **(g)** Quantitative analysis of ^{15}N/^{14}N signal per bacterium shown as violin plot with single dots. Grey line indicates the natural background level of the ^{15}N/^{14}N enrichment. Results were obtained from 264-439 individually segmented bacteria from n = 2 biologically independent experiments. **(h)** Quantification of the percentage of PZA/POA positive (PZA/POA^{+}) bacteria. Results are expressed as mean ± SEM from n = 2 biologically independent experiments. **(i)** Quantitative analysis of E2-Crimson Mtb WT and Mtb ΔRD1 area per single-cell expressed in µm^2. Results are shown in violin plots where grey lines represent the mean Mtb area per cell pre-treatment (t_{24h}) and black dots represent the mean Mtb area per cell post-treatment (t_{96h}). From 2272 to 2922 and 1331 to 3157 infected MDM were analysed for Mtb WT and Mtb ΔRD1 respectively. Results are from n = 2 biologically independent experiments. **(j)** Mean bacterial area per macrophage in the presence or absence of 100 nM ConA and increasing concentration of PZA was normalized and plotted as relative growth. Data was normalised with the mean Mtb area per cell pre-treatment (t_{24h}) and the control condition without PZA was used as reference corresponding to 100 % growth. Results are shown as the mean ± SEM from n = 2 biologically independent experiments performed in technical triplicate.

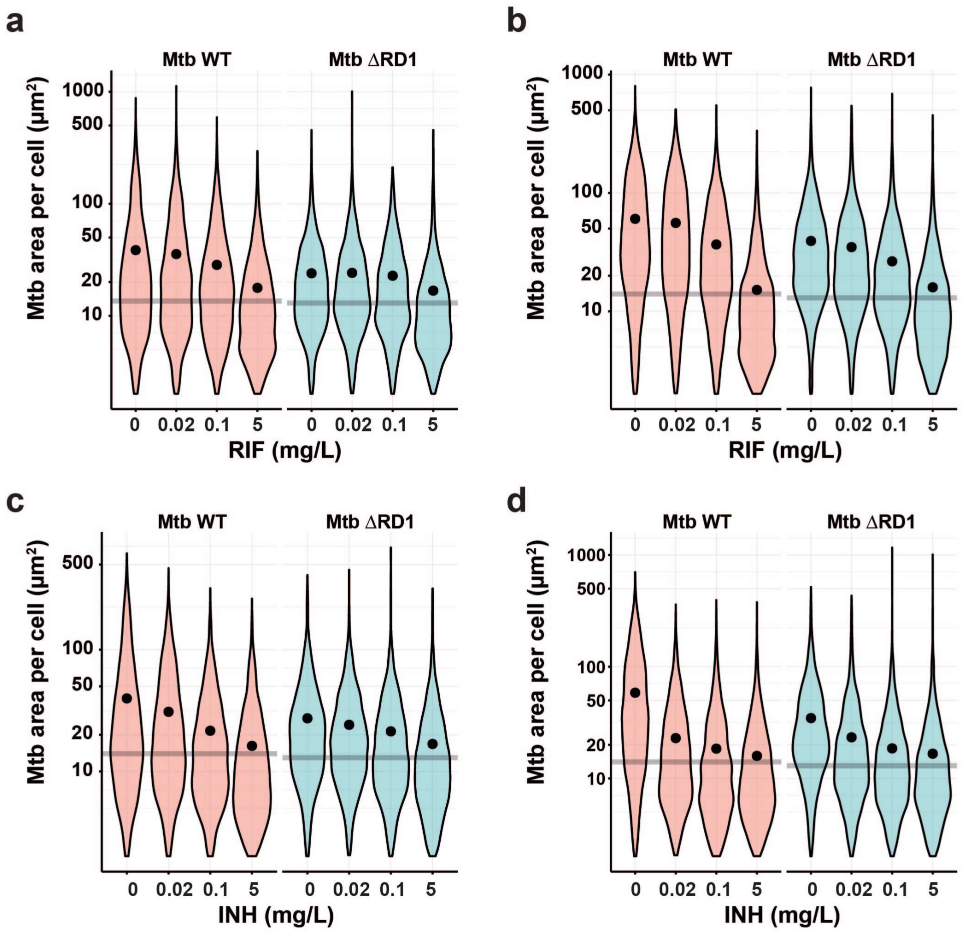

Supplementary Fig. 12 Inhibition of MDM endolysosomal acidification by BafA1 doesn't impact Mtb susceptibility towards other anti-TB frontlines drugs.

(a-d) Quantitative analysis of E2-Crimson Mtb WT and Mtb ΔRD1 area per single-cell expressed in μm^2 in the presence of increasing concentration of RIF (0-5 mg/L) or INH (0-5 mg/L). Results are displayed in violin plots where grey lines represent the mean Mtb area per cell pre-treatment (t_{24h}) and black dots represent the mean Mtb area per cell post-treatment (t_{96h}). **(a and c)** Antibiotic inhibition assays were performed in the absence of BafA1 whereas 100 nM BafA1 was used in the conditions depicted in **(b and d)**. From 2761 to 4536 and 2487 to 3791 infected MDM were analysed for Mtb WT and Mtb ΔRD1 respectively. Results are from n = 2 biologically independent experiments.

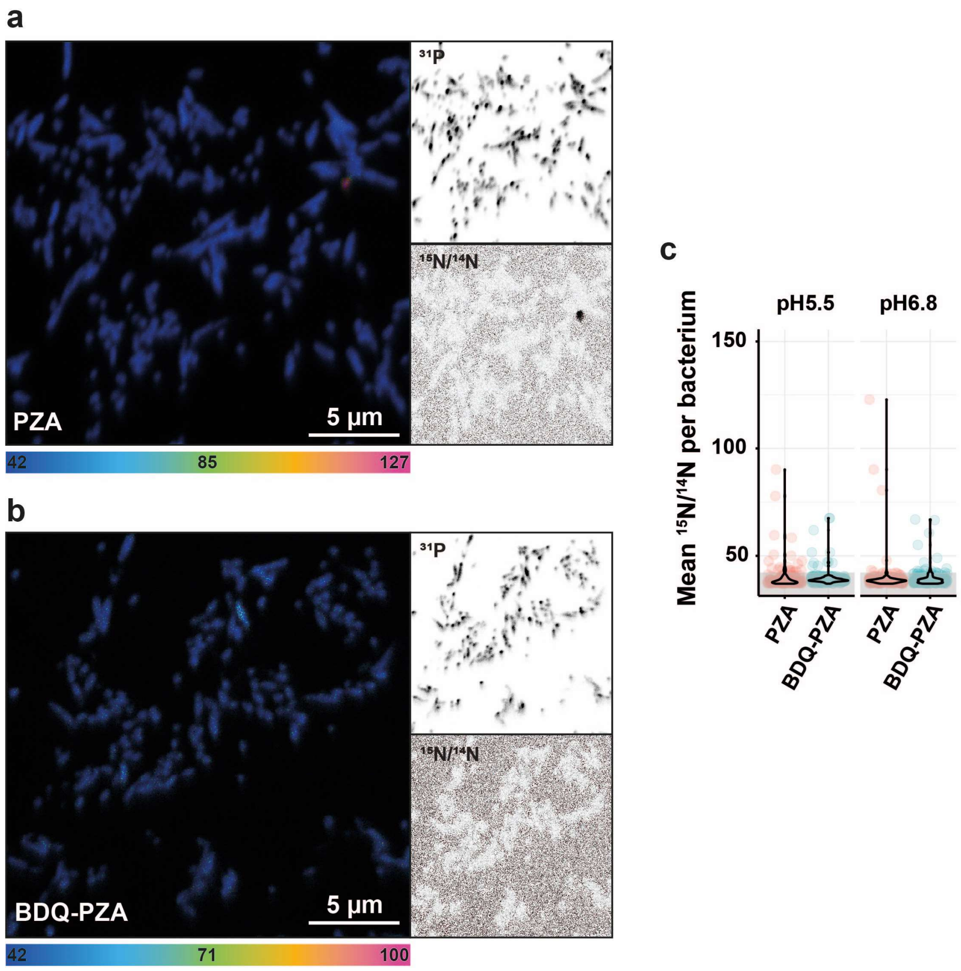

Supplementary Fig. 13 BDQ doesn't enhance PZA/POA accumulation *in vitro*

(a-b) Representative images of PZA/POA accumulation in Mtb WT *in vitro* in the presence of BDQ. Mtb WT was grown until reaching mid-exponentially growing phase in Middlebrook 7H9 medium, inoculated in fresh media adjusted at pH6.6 or pH5.5 and further treated with 30 mg/L [$^{15}N_2$, $^{13}C_2$]-PZA alone or in combination with 2.5 mg/L BDQ for 24 hours. Micrographs display $^{15}N/^{14}N$ signals as hyperspectral images. Magnifications show ^{31}P (top panel) and $^{15}N/^{14}N$ (bottom panel) individual NanoSIMS images. Micrographs are representative of one single experiment. **(c)** Quantitative analysis of $^{15}N/^{14}N$ signal per bacterium shown as violin plot with single dots. Grey line indicates the natural background level of the $^{15}N/^{14}N$ enrichment. In that specific context, bacterial segmentation was performed using the ^{31}P signal and the "threshold" function from FIJI. The "analyse particles" function from FIJI was used to quantify $^{15}N/^{14}N$ in each region of interest. Results are from 254-352 individual regions of interests from n = 1 biologically independent experiment.

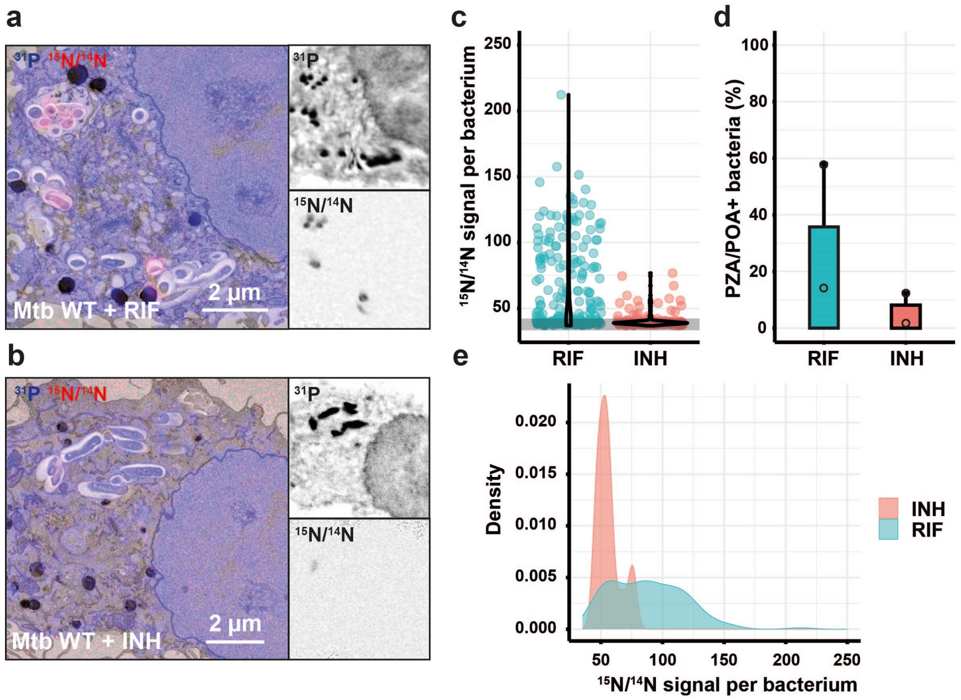

Supplementary Fig. 14 RIF but not INH enhances PZA/POA accumulation *in cellulo*

(a-b) Representative images of PZA/POA distribution in intracellular Mtb WT strains treated with RIF or INH. MDM were infected with Mtb WT treated with 30 mg/L [$^{15}N_2$, $^{13}C_2$]-PZA in the presence of 5mg/L of RIF or INH for 24 h. EM micrographs are overlaid with ^{31}P (blue) and $^{15}N/^{14}N$ (red) NanoSIMS images. Magnifications show ^{31}P (top panel) and $^{15}N/^{14}N$ (bottom panel) individual images at the single bacterial-cell level. Scale bars correspond to 2 µm. Micrographs are representative of two independent experiments. **(c)** Quantitative analysis of $^{15}N/^{14}N$ signal per bacterium shown as violin plot with single dots. Grey line indicates the natural background level of the $^{15}N/^{14}N$ enrichment. Results were obtained from 223-241 individually segmented bacteria from n = 2 biologically independent experiments. **(d)** Quantification of the percentage of PZA/POA positive (PZA/POA^{+}) bacteria. Results are shown as the mean ± SEM from n = 2 biologically independent experiments. **(e)** Analysis of the $^{15}N/^{14}N$ signal profile of Mtb WT PZA/POA positive bacteria in the presence of RIF or INH.
